# Subcutaneous implantable cardioverter defibrillator in patients awaiting cardiac transplantation or left ventricular assist device for refractory heart failure: a feasible alternative to transvenous device?

**DOI:** 10.1002/ehf2.12255

**Published:** 2018-01-27

**Authors:** Federico Migliore, Giacomo Cavalli, Tomaso Bottio, Pietro De Franceschi, Emanuele Bertaglia, Gino Gerosa, Sabino Iliceto

**Affiliations:** ^1^ Department of Cardiac, Thoracic and Vascular Sciences University of Padua Padua Italy

With an increasing population of patients with end‐stage heart failure (HF), orthotopic heart transplantation (OHT) remains an important selective treatment option. Moreover, despite donor pool expansion strategies, the unbalance between organ donors and patients needing OHT led to an increase in waiting‐list time; and thus, the rate of left ventricular assist device (LVAD) implantation, as bridge to either transplant or destination therapy, has more than doubled over the last 10 years.^1^

In order to prevent arrhythmic death and to improve cardiac performance in those awaiting cardiac transplantation, cardiac implantable electronic devices (CIEDs), including transvenous implantable cardioverter defibrillator (T‐ICD) and cardiac resynchronization therapy device, are recommended.^2^ Thus, both the population of patients with CIED who will undergo OHT and those with CIEDs/LVAD combination are destined to increase in the future years.

Interestingly, a significant portion of patients with CIEDs and advanced HF, ranging from 46% to 77%, does not have a strict pacing indication, including need of resynchronization therapy, as the main aim is primary prevention of sudden cardiac death.^3^, ^4^

The subcutaneous implantable cardioverter defibrillator (S‐ICD) was developed as an alternative therapy to T‐ICD system, as it is a fully subcutaneous system without any transvenous or epicardial leads.^5^, ^6^ Thus, the S‐ICD system has the potential to decrease periprocedural implantation risks, eliminate the problem of difficult venous access, reduce endovascular mechanical stress on leads, and decrease the risk of systemic device‐related infection typically observed in patients with a T‐ICD, even with the limit of lack of pacing capability as either antitachycardia pacing or sustained pacing for bradycardia. Therefore, S‐ICD could play an important clinical role in a significant portion of patients with HF.

Furthermore, OHT and LVAD patients with an ICD have some peculiar issues related to the presence of leads inside the cardiac chambers and the venous system, which we are going to address in this paper.

Concerning OHT, ICD leads are usually cut when superior vena cava section is performed during surgical operation, and the portion of the leads dwelling in the recipient's heart is removed from the organ. Then, the proximal portion of the leads is often extracted by manual traction. However, in a non‐negligible percentage of patients, ranging from 13% to 42%,^7^, ^8^ this procedure results in a not complete removal of the latter portion, owing to lead–tissue adhesion within the venous system. The most common site for these retained lead fragments is the central venous system (*Figure* 1 *A*). It has been described elsewhere^3^, ^4^ that the presence of retained ICD lead fragments after OHT would not impact long‐term morbidity and mortality. However, raising data reported different potential life‐threatening complications, including lead fragment infection, pulmonary embolization, venous obstruction, and electrical interferences with other CIEDs, ranging from 3.4% to 20%.^8^, ^9^ Currently, there are no data in the literature focused on S‐ICD patients who received OHT, with only sporadic cases reported in larger series: Pettit *et al*.^8^ reported a series of 206 patients with CIED at time of OHT, in which three (1%) had S‐ICD, who did not presented any early or late complication related to device removal. In addition, if needed, an implanted S‐ICD can be turned off, left in site at the time of OHT, and subsequently turned on again after testing sensor vectors, with no need to re‐implant another device (*Figure* 1 *B*).

**Figure 1:**
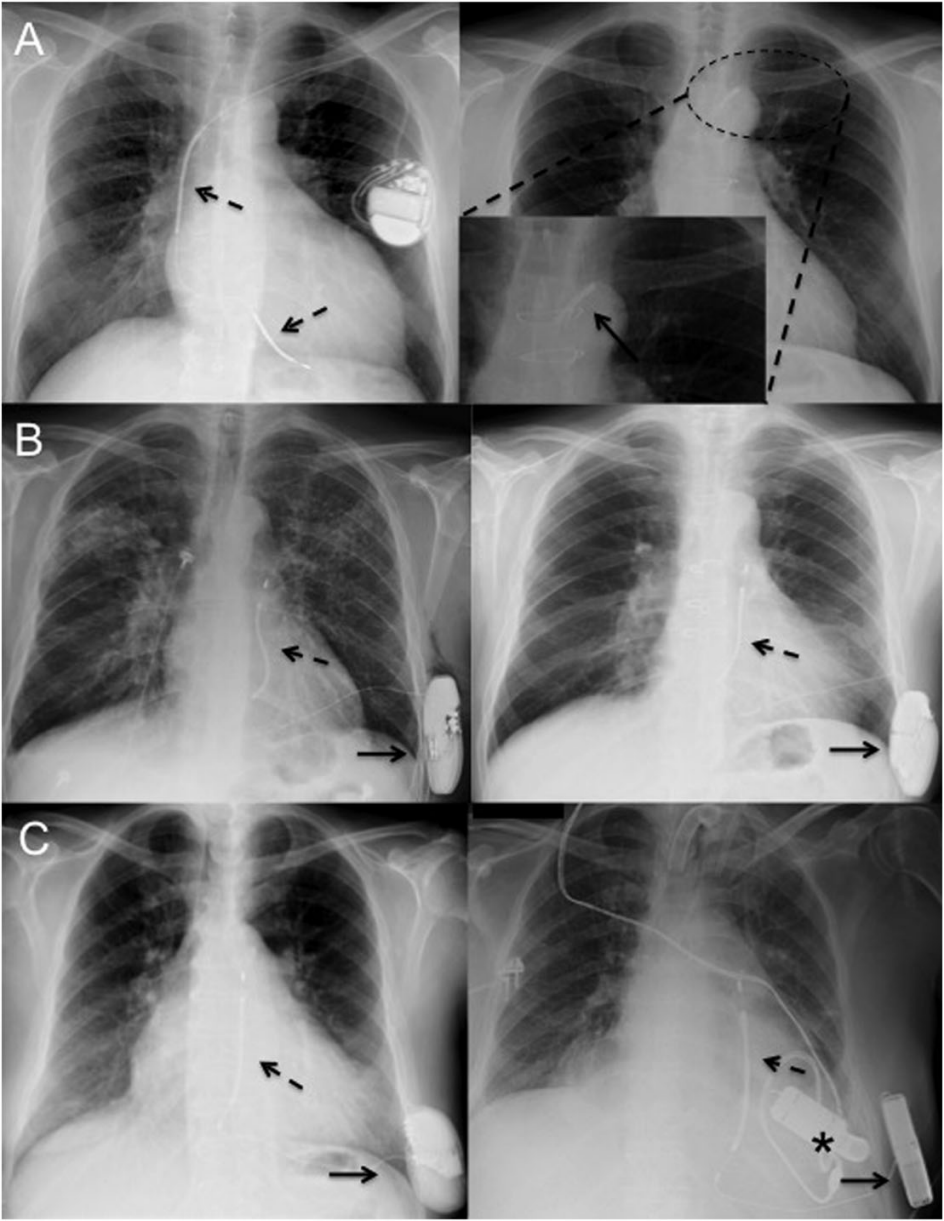
(A) Transvenous implantable cardioverter defibrillator with double‐coil lead (dashed arrows) dwelling in superior vena cava and right ventricle (left). Retained coil and lead (black arrow) fragment, after orthotopic cardiac transplantation in the same patient (right). (B) Subcutaneous lead (dashed arrow) and generator (black arrow) of a subcutaneous implantable cardioverter defibrillator in a patient with ischaemic heart disease and severe systolic dysfunction (left). Subcutaneous implantable cardioverter defibrillator after orthotopic heart transplantation in the same patient. The device interrogation revealed a stable sensing without interferences. (C) Subcutaneous lead (dashed arrow) and generator (black arrow) of a subcutaneous implantable cardioverter defibrillator in a patient with dilated cardiomyopathy and severe systolic dysfunction (left). Subcutaneous implantable cardioverter defibrillator after left ventricular assist device (Jarvik 2000, Jarvik Heart Inc.) positioning in left ventricular apex (asterisk) in the same patient (right). The device interrogation revealed a stable sensing without interferences.

Thus, we can easily affirm that at time of transplantation, the S‐ICD device can be either left in site at time of OHT or explanted in all its components, with a very low risk of major complication or infection, and virtually removing any potential complications due to retained lead fragments observed in T‐ICD patients.

Possible devices' interaction raises more challenging issues in patients with LVAD and T‐ICD. Different retrospective studies showed significant variation in sensing, lead impedance, pacing threshold, and device–device communication interactions in T‐ICD after LVAD implantation, with 13% of patients requiring lead revisions and 20% requiring ICD testing.^10^, ^11^ Moreover, electrical artefacts due to LVAD activity could cause inappropriate shock delivery in up to 18% of patients.^10^ The suspected mechanism is linked to LV unloading and to the concomitant leftward shifting of the septum after LVAD implantation, thus altering the ICD lead–myocardium conformation.^11^

If there is a general agreement about possible interaction between T‐ICD and LVAD, the precise role of S‐ICD in patients with LVAD (*Figure* 1 *C*) is not well established because of the paucity of data about the potential risk of interference leading to inappropriate shocks and the lack of data about the efficacy for the termination of spontaneous ventricular arrhythmias.^12^, ^13^ The inappropriate S‐ICD shocks may be due to electromagnetic interference, changes in R wave morphology, and decreasing R wave amplitude after LVAD implantation.^13^ Because the device automatically adjusts its sensing threshold to the amplitude of the last sensed events, post‐operative low voltage increases the risk of ventricular oversensing. However, there are emerging and encouraging data demonstrating that S‐ICD, if all sensing vectors before and after LVAD activation are correctly tested or its speed increases, is a safe and effective alternative to conventional ICD in terminating ventricular arrhythmias without evidence of mechanical interferences also with different types of LVAD.^12^, ^14^

A potential limitation of this novel technique could be a potential greater complexity for battery replacement. However, we believe that both the long battery duration of the current new‐generation S‐ICD system and reduction of acute and late complications may overcome this potential limitation.

Moreover, although a potential clinical limitation of the combination of S‐ICD and LVAD could be the inappropriate shocks due to interference, we believe that the advantages of the S‐ICD, including the lack of endocardial lead, and the low risk of lead failure and systemic infection, may overcome this limitation exactly in this particular kind of population.

In conclusion, taking into account these scenarios, it is evident that the implant of T‐ICD, a part of known early and late complications, implies a burden of specific and peculiar issues that could affect quality of life and overall survival in high‐risk patients like those awaiting OHT and with LVAD. Subcutaneous ICD is emerging as a promising option that could solve those aspects and therefore should be considered as a first choice in a significant portion of patients with HF and without indication for pacing, and not only as a second‐line therapy when a T‐ICD implant is not feasible or contraindicated.

## Conflict of interest

None declared.
